# Load distribution across weekly microcycles according to match schedule in a team competing in the Australian national A-League Women’s soccer competition

**DOI:** 10.5114/biolsport.2025.144413

**Published:** 2024-12-13

**Authors:** Aaron T. Scanlan, Dean Miller, Mia Lundquist, Nathan Elsworthy, Michele Lastella

**Affiliations:** 1 School of Health, Medical and Applied Sciences, Central Queensland University, Rockhampton, Queensland, Australia; 2 Appleton Institute for Behavioural Science, Central Queensland University, Adelaide, South Australia, Australia; 3 Adelaide United Football Club, Adelaide, South Australia, Australia

**Keywords:** Periodization, Women, Football, Workload, Microsensor, Global positioning

## Abstract

This observational, longitudinal study compared daily loads experienced in the weekly microcycle for different match schedules among an A-League Women’s professional soccer team. Monitoring data were retrospectively accessed from a team (n = 22) competing in the Australian National A-League Women’s soccer competition across the 2022–2023 in-season. Internal (session-rating of perceived exertion [session-RPE] and session-RPE load) and external load (total and relative values for total and high-speed running distance) data were acquired from 54 field-based training sessions and 17 matches across a 20-week period. Weeks were categorized according to schedule as: single-match week played on Saturday; single-match week played on Sunday; condensed week (6 days since the previous match); or double-header week (two matches in the same week). Sessions during each week were classified according to the day on which they were conducted prior to match day (MD) as MD-1, MD-2, MD-3, MD-4, MD-5, or MD-6. Linear mixed effects models and Hedge’s g_av_ effect sizes were used to compare variables between days. All load variables were highest on MD (P < 0.001, g_av_ = 0.36–7.84, small-to-very large), with the day before matches being generally lower than other training days across schedules (g_av_ = 0.01–3.89, trivial-to-very large). Further, an extra training day was prescribed in single-match weeks when played on Sunday compared to Saturday, with training microcycles appearing rather consistent across weekly schedules. These data may be used as an initial reference for practitioners working in this competition or women’s soccer settings. The relatively similar daily periodization patterns across different match schedules highlight greater consideration of weekly match schedules may be needed when planning weekly microcycles according to the schedule congestion faced.

## INTRODUCTION

Women’s soccer is relishing a period of unprecedented popularity and media attention in Australia [1], culminating in being a joint host nation for the 2023 FIFA Women’s World Cup. In this regard, participation in soccer among females across all ages in Australia has increased by approximately 45% from ~412,000 in 2016 to ~596,000 in 2022, with females now representing 28% of participants [2]. Despite the growth of women’s soccer in Australia, sport science research dedicated to this population on a global scale are not comparable to their male counterparts [3]. For instance, bibliometric analyses showed that 22% of soccer studies across various applied sport science topics published between 2010–2019 included female participants, which decreases to 15% at the professional level [3]. Moreover, only 12% of studies encompassing load monitoring among professional soccer players examined females across this timeframe [3].

Load monitoring is a fundamental area in applied sport science research, used to describe the locomotor characteristics and understand the demands imposed on athletes within a given context [4]. Load data can assist in planning the training process, informing decisions regarding any necessary adjustments in managing teams and individual players across desired cycles [5]. In this way, loads are commonly planned across microcycles during the season, varying from day-to-day within each week to best balance physical preparation and readiness for upcoming matches among players [5, 6]. Survey data acquired from sport science practitioners working with professional soccer teams indicate recovery sessions are predominantly implemented in the two days following matches, with acquisition strategies such as aerobic and resistance training implemented to varying degrees across the next two days, and skill/tactical training performed from mid-week to the day before matches [7]. Indeed, studies examining professional, female soccer players from various competitions in Europe [8–11] support this trend showing a peak in loading around mid-week following recovery days, then a gradual decline in load leading into the next match. These data form useful reference evidence bases concerning the periodization schemes adopted in teams competing at the highest levels in female soccer.

Despite research quantifying the distribution of daily loads across weekly microcycles among professional, female soccer players, existing data are not indicative of professional competition in Australia, nor does it consider match scheduling. In this way, the competition being investigated has been shown to impact the demands experienced during matches in professional, male soccer players [12]. Furthermore, different competitions are likely to impose unique match scheduling, which may impact the weekly loading strategies adopted by teams [5]. Within an Australian context, few studies have quantified the loads experienced among professional, female soccer players competing in the Australian national A-League Women’s competition. Specifically, the movement profiles [13], total and highspeed running (HSR) distances [13–15], repeated high-intensity demands [16], and acceleration demands [14, 17] experienced during matches have been reported for this population, with no studies comprehensively quantifying the loads experienced during training. Moreover, the available research is indicative of the A-League Women’s competition from five or more years ago meaning an account of contemporary load data is lacking, which is important given the temporal changes in demands that can occur within soccer competitions [18]. Consequently, exploration of loading across weekly microcycles specifically within the A-League Women’s competition will provide an initial reference regarding the typical daily loads and periodization schemes experienced in a team context for practitioners working in this competition, such as coaches, strength and conditioning specialists, and sport scientists. More widely, these data may provide useful insight into the strategies adopted to manage training loads across the week considering the match schedule being faced within professional, female soccer settings.

Consequently, this study aimed to examine the daily load distribution across weekly microcycles in the A-League Women’s Australian national soccer competition in consideration of match scheduling. Based on the documented approaches adopted by sport science practitioners [7] and monitoring data provided for professional, female soccer players competing in other leagues [8–11], we hypothesised that daily loading would be highest on match days (MD) and mid-week, with reduced loading following matches and leading into matches. As a novel line of inquiry in professional, female soccer players, we also hypothesised that loading periodization would be relatively consistent across different weekly match schedules given this pattern has been readily observed in monitoring research examining male soccer teams [19–21].

## MATERIALS AND METHODS

### Study design

A retrospective, observational, longitudinal study design was adopted whereby a single team of professional, female soccer players were monitored during field-based training sessions and matches across a season.

### Subjects

Players (n = 22; age: 22.5 ± 3.7 years; height: 170 ± 7 cm; body mass: 65.3 ± 7.7 kg) from the same team competing in the A-League Women’s competition participated. These players consisted of nine defenders, six midfielders, and seven forwards. Players were included in the study if they were registered members of the monitored team and participated in any field-based team training sessions and/or matches throughout the season. Players occupying goalkeeper positions were excluded from our study given the unique demands they experience during training and match settings as commonly adopted in monitoring research [8–10]. Retrospective access to the acquired data for this study was granted by the Central Queensland University Human Research Ethics Committee (#0000024246) with all procedures performed in accordance with the Helsinki Declaration [22].

### Training and match schedule

**FIG. 1:**
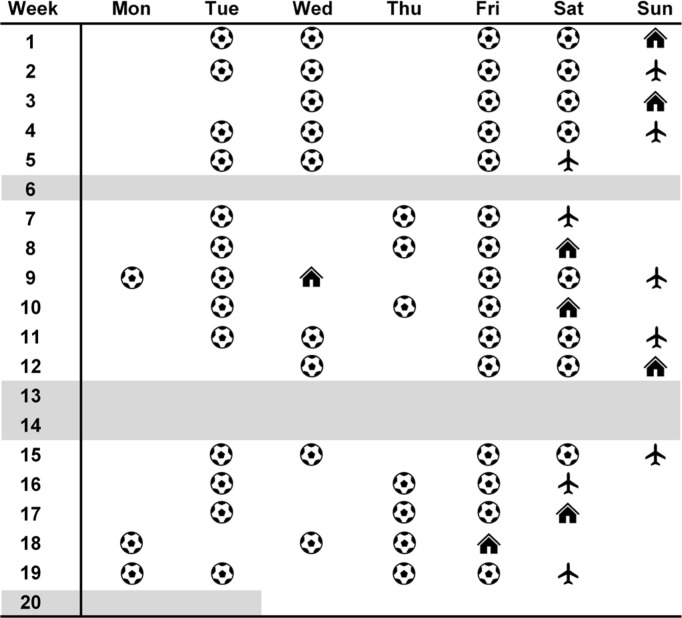
The in-season schedule of the team investigated in this study. *Note*: soccer ball symbol represents field training sessions monitored; house symbol represents matches played at home; plane symbol represents matches played away; weeks shaded in grey were not analyzed due to irregular scheduling in which no matches were played (weeks 6, 13, and 14) or the final match was played on a Tuesday (week 20).

The 20-week in-season phase during the 2022–2023 season lasted from November to March, with 54 field-based training sessions and 17 matches (eight at home and nine away) included in this study across this period. The full in-season schedule is shown in Figure 1. Monitored matches took place on either a Wednesday (*n* = 1), Friday (*n* = 1), Saturday (*n* = 8), or Sunday (*n* = 7) in each week. Accordingly, weeks were categorized according to their scheduling based on the potential to induce varied daily loading schemes across the week including: (1) a single-match week played on Saturday with at least seven days since the previous match (*n* = 5 weeks); (2) singlematch week played on Sunday with at least seven days since the previous match (*n* = 7 weeks); (3) condensed week where a match was played on a Friday or Saturday with only six days since the previous match (played on a Saturday or Sunday in the prior week) (*n* = 3 weeks); or (4) double-header week where two matches were played in the same week on Wednesday and Sunday (*n* = 1 week). In turn, field-based training sessions were generally held on days related to MD in each week. In this way, field-based training sessions were identified as occurring on one (MD-1), two (MD-2), three (MD-3), four (MD-4), five (MD-5), or six (MD-6) days prior to the match each week. In condensed and double-header weeks, the most recent match was identified as MD, with the earlier match identified on MD-6 (in the condensed weeks) or MD-4 (in the double-header week). Importantly, there were three weeks in which no matches were played (i.e., bye weeks), which were excluded from our analyses given the inconsistent training scheduling and player availability evident, with a predominant team focus on recovery and maintenance in these weeks. Moreover, the final week was excluded from analyses given data were not captured in the final match (played on a Tuesday at home), which was irregularly scheduled three days following the previous match. Overall, 1,044 daily observations were gathered in this study, with the number of observations for each day in each week category shown in Table 1.

**TABLE 1:** Mean ± standard deviation daily internal and external load variables across weekly microcycles according to in-season match schedule in a team competing in the Australian national A-League Women’s soccer competition.

Match schedule and variable	Day						

	MD-6	MD-5	MD-4	MD-3	MD-2	MD-1	MD
**Single-match week on Saturday (n)**	‡	†	58	†	57	60	50
Duration (min)	-	-	71.6 ± 7.8	-	74.0 ± 9.2	62.6 ± 12.0^c,e^	77.2 ± 33.0^f^
Session-RPE (AU)	-	-	3.7 ± 0.6	-	3.5 ± 0.7	3.1 ± 0.5^e^	7.5 ± 1.5^c,e,f^
Session-RPE load (AU)	-	-	266 ± 59	-	253 ± 39	195 ± 44	620 ± 303^c,e,f^
Total distance (m)	-	-	5127 ± 1076	-	5025 ± 553	3128 ± 603^c,e^	7938 ± 3312^c,e,f^
Relative total distance (m · min^−1^)	-	-	71.5 ± 11.4	-	68.5 ± 8.2	50.4 ± 7.4^c,e^	104.5 ± 9.1^c,e,f^
HSR distance (m)	-	-	308 ± 132	-	275 ± 80	73 ± 39^c,e^	574 ± 265^c,e,f^
Relative HSR distance (m · min^−1^)	-	-	4.4 ± 1.9	-	3.8 ± 1.1	1.2 ± 0.6^c,e^	8.1 ± 3.1^c,e,f^

**Single-match week on Sunday (n)**	‡	67	110	‡	111	108	95
Duration (min)	-	66.8 ± 7.8	68.4 ± 11.4	-	68.3 ± 8.5	58.4 ± 3.6^b,c,e^	77.1 ± 32.2^b,c,e,f^
Session-RPE (AU)	-	3.5 ± 0.8	3.6 ± 0.6	-	3.4 ± 0.7	3.0 ± 0.4^b,c,e^	7.2 ± 1.4^b,c,e,f^
Session-RPE load (AU)	-	235 ± 64	253 ± 60	-	232 ± 43	178 ± 29^b,c,e^	589 ± 284^b,c,e,f^
Total distance (m)	-	4336 ± 730	4808 ± 931	-	4439 ± 669	2934 ± 581^b,c,e^	7777 ± 3427^b,c,e,f^
Relative total distance (m · min^−1^)	-	64.9 ± 7.5	70.5 ± 9.3^b^	-	65.2 ± 8.8^c^	50.4 ± 10.1^b,c,e^	101.1 ± 16.7^b,c,e,f^
HSR distance (m)	-	92 ± 58	308 ± 169^b^	-	252 ± 97^b,c^	73 ± 47^c,e^	565 ± 288^b,c,e,f^
Relative HSR distance (m · min^−1^)	-	1.4 ± 0.8	4.5 ± 2.5^b^	-	3.7 ± 1.4^b,c^	1.3 ± 0.8^c,e^	7.8 ± 3.1^b,c,e,f^

**Condensed week (n)**	51*	‡	51	‡	42	58	52
Duration (min)	78.6 ± 32.8	-	69.5 ± 4.1	-	71.3 ± 7.0	54.9 ± 3.7^a,c,e^	75.2 ± 34.5^f^
Session-RPE (AU)	7.5 ± 1.7	-	3.4 ± 0.6^a^	-	3.4 ± 0.5^a^	2.9 ± 0.6^a,e^	7.9 ± 1.3^c,e,f^
Session-RPE load (AU)	626 ± 301	-	238 ± 48^a^	-	241 ± 29^a^	161 ± 30^a^	621 ± 311^c,e,f^
Total distance (m)	8053 ± 3338	-	3947 ± 973^a^	-	4870 ± 469^a^	3075 ± 450^a,e^	7749 ± 3437^c,e,f^
Relative total distance (m · min^−1^)	103.4 ± 9.81	-	56.6 ± 12.8^a^	-	68.5 ± 4.9^a,c^	56.1 ± 8.2^a,e^	104.8 ± 8.8^c,e,f^
HSR distance (m)	558 ± 270	-	79 ± 70^a^	-	307 ± 89^a,c^	83 ± 44^a,e^	549 ± 314^c,e,f^
Relative HSR distance (m · min^−1^)	7.5 ± 2.7	-	1.1 ± 0.9^a^	-	4.3 ± 1.4^a,c^	1.5 ± 0.7^a,e^	7.8 ± 3.3^c,e,f^

**Double-header week (n)**	14	13	14*	‡	13	14	12
Duration (min)	79.1 ± 0.0	51.2 ± 0.0^a^	69.4 ± 33.8	-	61.4 ± 0.0	59.3 ± 0.0^a^	72.7 ± 31.2^b^
Session-RPE (AU)	3.3 ± 0.6	2.7 ± 0.5	7.8 ± 1.5^a,b^	-	3.9 ± 0.4^b,c^	3.0 ± 0.1^c,e^	7.8 ± 1.5^a,b,e,f^
Session-RPE load (AU)	260 ± 46	136 ± 24	570 ± 316^a,b^	-	241 ± 28^c^	180 ± 8^c^	598 ± 291^a,b,e,f^
Total distance (m)	4425 ± 408	2685 ± 355	7409 ± 3517^a,b^	-	3252 ± 241^c^	3313 ± 271^c^	7430 ± 3031^a,b,e,f^
Relative total distance (m · min^−1^)	56.0 ± 5.2	52.4 ± 6.9	107.7 ± 9.0^a,b^	-	53.0 ± 3.9^c^	55.9 ± 4.6^c^	103.7 ± 8.9^a,b,e,f^
HSR distance (m)	132 ± 76	54 ± 31	588 ± 302^a,b^	-	183 ± 34^c^	132 ± 41^c^	526 ± 231^a,b,e,f^
Relative HSR distance (m · min^−1^)	1.7 ± 1.0	1.0 ± 0.6	8.9 ± 3.1^a,b^	-	3.0 ± 0.6^b,c^	2.2 ± 0.7^c^	7.5 ± 1.6^a,b,e,f^

*Abbreviations*: MD, match day; MD-1, day before match day; MD-2, 2 days before match day; MD-3, 3 days before match day; MD-4, 4 days before match day; MD-5, 5 days before match day; MD-6, 6 days before match day; *n*, number of player samples for that day within that match schedule; RPE, rating of perceived exertion; AU, arbitrary units; HSR, high-speed running.

*Note*:

‡ represents days on which no sessions were held across the season;

† represents days where only one session was held across the season and therefore excluded from analyses;

* indicates matches were played on these days; superscripted letters indicate statistically significant differences between days within that weekly match schedule (*P* < 0.05) where

a indicates different to MD-6,

b indicates different to MD-5,

c indicates different to MD-4,

d indicates different to MD-3,

e indicates different to MD-2, and

f indicates different to MD-1.

### Procedures

The Strengthening the Reporting of Observational Studies in Epidemiology (STROBE) statement [23] was followed in developing this manuscript. To obtain baseline descriptive anthropometric data for all players, stature and body mass were measured using dual-energy x-ray absorptiometry scans (Horizon Wi (S/N304832M); Hologic; Macquarie Park, Australia) at the commencement of the in-season phase.

Players had their internal load (i.e., responses to the exercise) monitored via the provision of individualized session-RPE scores using Foster’s modified CR-10 scale acquired in the presence of only the investigator 30 minutes following each training session and match [24]. Session-RPE data were then multiplied by the duration (in minutes) of the corresponding session to provide session-RPE load in arbitrary units (AU) [24]. Training session durations (for calculation of session-RPE load and determination of GPS variables) were recorded from the commencement of the team warm-up given it was prescribed as part of the training stimuli until the completion of the final drill [11]. Match durations (for calculation of session-RPE load and determination GPS variables) were taken as the time in which each player was competing between the start and end of the match, excluding any time on the bench and during the half-time break [25].

Players had their external load (i.e., physical work performed) routinely monitored by sport science staff using Catapult PlayerTek™ GPS devices sampling at 10 Hz (PlayerTek™ Pod; Catapult Sports; Melbourne, Australia) affixed to the torso via accompanying neoprene vests worn under playing attire. Devices were switched on sufficiently prior to warm-up for each training session and match to ensure adequate satellite connection [14]. These devices have been previously used to monitor external loads in female soccer players [9, 25], with research [26] supporting their validity (total distance vs. manual distance measurement = < 0.01%, *P* > 0.05; peak speed vs. radar gun speed = 0.03%, *P* > 0.05) and retest reliability (coefficient of variation = 1.1% for total distance to 11.7% for very highspeed distance [> 22 km · h^−1^]) during running and sprinting tasks in field-based team sport settings. Each player wore the same device throughout the study to account for any potential inter-device variability in outputs. Data were uploaded from each device following training sessions and matches (oneapp.catapultsports.com) before being processed using proprietary software (PlayerTek™ Cloud; Catapult Sports; Melbourne, Australia). Outputs for each player during training sessions and matches were trimmed according to relevant durations using the proprietary software via recorded times and visual inspection [27]. Data summaries were then exported into a Microsoft Excel spreadsheet (v15, Microsoft Corp; Redmond, WA). Data obtained in individual training sessions from players who did not complete the entire session or were undergoing adjusted training loads due to injury were excluded from our analyses [10]. There was no minimum playing time set for inclusion of match data in our analyses with 93% of our match observations involving players participating for > 20 minutes. Various external load variables were determined including total distance (m), relative total distance (m · min^−1^), high-speed running (HSR) distance (> 18 km · h^−1^ in m), and relative HSR distance (> 18 km · h^−1^ in m · min^−1^). Relative variables were determined as the total values divided by the duration across which they were accumulated. The threshold for HSR distance aligns with previous research quantifying high-intensity running demands in professional, female soccer players [28] and seemed practically applicable given it was routinely adopted by coaching staff when monitoring the sample of players we investigated.

### Statistical analyses

Data were imported from Microsoft Excel into RStudio (v4.1.3; R Core Team) for cleaning and analyses. The dataset was compiled in long form with each row representing an observation and load variables presented in columns. Separate linear mixed effects models were utilized to assess differences in external and internal load variables between days. Customized script was developed to undertake analyses and is provided as a supplementary file. Models were built using the *lme4* [29] package in RStudio. Firstly, a model with all fixed and random effects was developed, along with a second model containing an interaction between day and schedule. These models were compared to a separate model, which was developed for each match schedule category. Following recommendations [30], the separated model was found to be the most suitable as indicated by the lowest Akaike information criteria value. In turn, separate models were developed according to match schedule (Saturday, Sunday, condensed, and double-header) and within each model, day (MD, MD-1, MD-2, MD-3, MD-4, MD-5, and MD-6) was entered as a fixed effect. In turn, player and week (i.e., competition round) were entered as random effects to account for repeated measures from individual players and match-to-match variations in load variables [30]. Following model development, histograms and Q-Q plots were produced from the residuals and visually inspected for normality. No statistical assumptions for using linear mixed effects models were violated for any variable, supporting this approach for all analyses. Pairwise comparisons were assessed post hoc with Tukey’s Honest Significant Difference between days within each match schedule using the *multcomp* package. Using a published spreadsheet [31], Hedge’s *g*_av_ was calculated to represent effect sizes in pairwise comparisons between days within each match schedule and interpreted as: *trivial* = < 0.20; *small* = 0.20–0.59; *moderate* = 0.60–1.19; *large* = 1.20–1.99; or *very large* = > 2.00 [32]. Descriptive data were calculated as mean ± standard deviation (SD) for all variables with significance set at *P* ≤ 0.05 for statistical analyses.

## RESULTS

The mean ± SD for all load variables on each day within each weekly match schedule category are shown in Table 1. *P*-values and Hedges *g*_av_ statistics for all pairwise comparisons between days within each weekly match schedule are shown in Table 2 for duration and internal load variables and in Table 3 for external load variables.

**TABLE 2:** *P*-values and Hedge’s *g*_av_ for pairwise comparisons in duration and internal load variables between days according to weekly match schedule in a team competing in the Australian national A-League Women’s soccer competition.

Match schedule		P-value, Hedge’s g_av_ for pairwise comparisons between days							Match schedule
**Duration**		MD-6	MD-5	MD-4	MD-2	MD-1	MD	
Single-match week on Saturday	MD-5	-	-	0.981, 0.17	0.989, 0.19	**0.003, 1.38**	**< 0.001, 0.44**	MD-5	Single-match week on Sunday
	MD-4	-	-	-	1.000, 0.01	**< 0.001, 1.19**	**< 0.001, 0.36**	MD-4	
	MD-2	-	-	0.943, 0.28	-	**< 0.001, 1.52**	**< 0.001, 0.37**	MD-2	
	MD-1	-	-	**0.021, 0.88**	**0.002, 1.06**	-	**< 0.001, 0.82**	MD-1	
	MD	-	-	**< 0.001, 1.14**	**< 0.001, 1.22**	**< 0.001, 2.01**	-	MD	

Condensed week		MD-6	MD-5	MD-4	MD-2	MD-1	MD		Double-header week
	MD-6	-	**< 0.001, NP**	0.686, 0.40	0.095, NP	**0.038, NP**	0.931, 0.29	MD-6	
	MD-5	-	-	0.086, 0.75	0.699, NP	0.840, NP	**0.034, 0.97**	MD-5	
	MD-4	0.338, 0.39	-	-	0.849, 0.33	0.674, 0.42	0.998, 0.10	MD-4	
	MD-2	0.569, 0.31	-	1.000, 0.31	-	1.000, NP	0.620, 0.51	MD-2	
	MD-1	**< 0.001, 1.01**MD-1	-	**0.001, 3.75**	**< 0.001, 2.93**	-	0.420, 0.60	MD-1	
	MD	0.955, 0.10	-	0.855, 0.23	0.961, 0.16	**< 0.001, 0.83**	-	MD	

*Session-RPE*		MD-6	MD-5	MD-4	MD-2	MD-1	MD		

Single-match week on Saturday	MD-5	-	-	0.670, 0.22	0.999, 0.09	**0.009, 0.69**	**< 0.001, 3.18**	MD-5	Single-match week on Sunday
	MD-4	-	-	-	0.320, 0.34	**< 0.001, 1.11**	**< 0.001, 3.20**	MD-4	
	MD-2	-	-	0.594, 0.37	-	**0.004, 0.67**	**< 0.001, 3.36**	MD-2	
	MD-1	-	-	**0.003, 0.98**	0.202, 0.57	-	**< 0.001, 3.89**	MD-1	
	MD	-	-	**< 0.001, 3.24**	**< 0.001, 3.44**	**< 0.001, 3.83**	-	MD	

Condensed week		MD-6	MD-5	MD-4	MD-2	MD-1	MD		Double-header week
	MD-6	-	0.389, 1.18	**< 0.001, 4.00**	0.432, 1.22	0.968, 0.59	**< 0.001, 4.08**	MD-6	
	MD-5	-	-	**< 0.001, 4.67**	**0.003, 2.72**	0.863, 1.09	**< 0.001, 4.75**	MD-5	
	MD-4	**< 0.001, 3.31**	-	-	**< 0.001, 3.53**	**< 0.001, 4.53**	1.000, 0.03	MD-4	
	MD-2	**< 0.001, 3.34**	-	0.359, 0.03	-	0.088, 2.65	**< 0.001, 3.61**	MD-2	
	MD-1	**< 0.001, 3.63**	-	0.232, 0.72	**< 0.001, 0.80**	-	**< 0.001, 4.61**	MD-1	
	MD	0.183, 0.31	-	**< 0.001, 4.54**	**< 0.001, 4.63**	**< 0.001, 4.93**		MD	

*Session-RPE load*		MD-6	MD-5	MD-4	MD-2	MD-1	MD		
Single-match week on Saturday	MD-5	-	-	0.946, 0.29	0.998, 0.06	**0.026, 1.14**	**< 0.001, 1.72**	MD-5	Single-match week on Sunday
	MD-4	-	-	-	0.739, 0.40	**< 0.001, 1.58**	**< 0.001, 1.64**	MD-4	
	MD-2	-	-	0.989, 0.25	-	**< 0.019, 1.47**	**< 0.001, 1.76**	MD-2	
	MD-1	-	-	0.052, 1.36	0.171, 1.39	-	**< 0.001, 2.03**	MD-1	
	MD	-	-	**< 0.001, 1.62**	**< 0.001, 1.70**	**< 0.001, 1.96**	-	MD	

Condensed week		MD-6	MD-5	MD-4	MD-2	MD-1	MD		Double-header week
	MD-6	-	0.375, 3.35	**< 0.001, 1.36**	0.999, 0.50	0.798, 2.41	**< 0.001, 1.61**	MD-6	
	MD-5	-	-	**< 0.001, 1.92**	0.618, 4.00	0.983, 2.42	**< 0.001, 2.22**	MD-5	
	MD-4	**< 0.001, 1.81**	-	-	**< 0.001, 1.46**	**< 0.001, 1.73**	0.999, 0.09	MD-4	
	MD-2	**< 0.001, 1.74**	-	**< 1.000, 0.39**	-	0.945, 2.96	**< 0.001, 1.71**	MD-2	
	MD-1	**< 0.001, 2.17**	-	0.122, 1.87	0.060, 2.82	-	**< 0.001, 2.01**	MD-1	
	MD	1.000,0.02	-	**< 0.001, 1.73**	**< 0.001, 1.67**	**< 0.001, 2.08**	-	MD	

*Abbreviations*: MD, match day; MD-1, day before match day; MD-2, 2 days before match day; MD-3, 3 days before match day; MD-4, 4 days before match day; MD-5, 5 days before match day; MD-6, 6 days before match day; RPE, rating of perceived exertion.

*Note*: Shaded cells show comparisons between days for Sunday and Double-header match schedules; bolded data indicate significant pairwise comparisons (*P* < 0.05); NP indicates the standard deviation could not be provided given all players had the same duration.

**TABLE 3:** *P*-values and Hedge’s *g*_av_ for pairwise comparisons in external load variables between days according to weekly match schedule in a team competing in the Australian national A-League Women’s soccer competition.

Match schedule		*P*-value, Hedge’s g_av_ for pairwise comparisons between days							Match schedule
**Total distance**		MD-6	MD-5	MD-4	MD-2	MD-1	MD		
Single-match week on Saturday	MD-5	-	-	0.331, 0.56	0.997, 0.15	**< 0.001, 2.12**	**< 0.001, 1.39**	MD-5	Single-match week on Sunday
	MD-4	-	-	-	0.389, 0.45	**< 0.001, 2.41**	**< 0.001, 1.18**	MD-4	
	MD-2	-	-	0.992, 0.12	-	**< 0.001, 2.40**	**< 0.001, 1.35**	MD-2	
	MD-1	-	-	**< 0.001, 2.28**	**< 0.001, 3.27**	-	**< 0.001, 1.97**	MD-1	
	MD	-	-	**< 0.001, 1.14**	**< 0.001, 1.22**	**< 0.001, 2.01**	-	MD	

		MD-6	MD-5	MD-4	MD-2	MD-1	MD		
Condensed week	MD-6	-	0.151, 4.51	**< 0.001, 1.18**	0.573, 3.47	0.614, 3.18	**< 0.001, 1.38**	MD-6	Double-header week
	MD-5	-	-	**< 0.001, 1.87**	0.974, 1.86	0.954,1.97	**< 0.001, 2.18**	MD-5	
	MD-4	**< 0.001, 1.67**	-	-	**< 0.001, 1.65**	**< 0.001, 1.63**	1.000, 0.01	MD-4	
	MD-2	**< 0.001, 1.33**	-	0.237, 1.20	-	1.000, 0.23	**< 0.001, 1.92**	MD-2	
	MD-1	**< 0.001, 2.08**	-	0.155, 1.15	**< 0.001, 3.89**	-	**< 0.001, 1.89**	MD-1	
	MD	0.952, 0.09	-	**< 0.001, 1.50**	**< 0.001, 1.17**	**< 0.001, 1.90**	-	MD	

**Relative total distance**		MD-6	MD-5	MD-4	MD-2	MD-1	MD		
Single-match week on Saturday	MD-5	-	-	**0.005, 0.66**	0.998, 0.04	**< 0.001, 1.62**	**< 0.001, 2.80**	MD-5	Single-match week on Sunday
	MD-4	-	-	-	**0.003, 0.58**	**< 0.001, 2.07**	**< 0.001, 2.27**	MD-4	
	MD-2	-	-	0.203, 0.27	-	**< 0.001, 1.56**	**< 0.001, 2.69**	MD-2	
	MD-1	-	-	**< 0.001, 2.00**	**< 0.001, 2.31**	-	**< 0.001, 3.68**	MD-1	
	MD	-	-	**< 0.001, 2.96**	**< 0.001, 4.15**	**< 0.001, 6.51**	-	MD	

		MD-6	MD-5	MD-4	MD-2	MD-1	MD		
Condensed week	MD-6	-	0.663,0.57	**<0.001,7.01**	0.572,0.64	1.000,0.02	< 0.001,6.53	MD-6	Double-header week
	MD-5	-	-	< 0.001,6.84	1.000,0.10	0.701,0.58	< 0.001,6.39	MD-5	
	MD-4	**< 0.001, 4.08**	-	-	< 0.001,7.84	< 0.001,7.22	0.510,0.44	MD-4	
	MD-2	**< 0.001, 4.49**	-	**< 0.001, 1.21**	-	0.610,0.66	< 0.001,7.34	MD-2	
	MD-1	**< 0.001, 5.21**	-	1.000, 0.05	**< 0.001, 1.82**	-	< 0.001,6.73	MD-1	
	MD	0.880, 0.15	-	**< 0.001, 4.37**	**< 0.001, 5.11**	**< 0.001, 5.72**	-	MD	

**HSR distance**		MD-6	MD-5	MD-4	MD-2	MD-1	MD	Day
Single-match week on Saturday	MD-5	-	-	**< 0.001, 1.70**	**< 0.001, 1.99**	0.854, 0.36	**< 0.001, 2.27**	MD-5	Single-match week on Sunday
	MD-4	-	-	-	**0.031, 0.40**	**< 0.001, 1.89**	**< 0.001, 1.09**	MD-4	
	MD-2	-	-	0.620, 0.30	-	**< 0.001, 2.33**	**< 0.001, 1.45**	MD-2	
	MD-1	-	-	**< 0.001, 2.42**	**< 0.001, 3.21**	-	**< 0.001, 2.38**	MD-1	
	MD	-	-	**< 0.001, 1.26**	**< 0.001, 1.52**	**< 0.001, 2.64**	-	MD	

		MD-6	MD-5	MD-4	MD-2	MD-1	MD		
Condensed week	MD-6	-	0.770, 1.35	**< 0.001, 2.05**	0.965, 0.86	1.000, 0.01	**< 0.001, 2.28**	MD-6	Double-header week
	MD-5	-	-	**< 0.001, 2.47**	0.285, 3.95	0.785, 2.11	**< 0.001, 2.85**	MD-5	
	MD-4	**< 0.001, 2.43**	-	-	**< 0.001, 1.87**	**< 0.001, 2.10**	0.909, 0.23	MD-4	
	MD-2	**< 0.001, 1.25**	-	**< 0.001, 2.84**	-	0.959, 1.35	**< 0.001, 2.06**	MD-2	
	MD-1	**< 0.001, 2.45**	-	1.000, 0.08	**< 0.001, 3.18**	-	**< 0.001, 2.36**	MD-1	
	MD	1.000, 0.03	-	**< 0.001, 2.06**	**< 0.001, 1.05**	**< 0.001, 2.07**	-	MD	

**Relative HSR distance**		MD-6	MD-5	MD-4	MD-2	MD-1	MD		
Single-match week on Saturday	MD-5	-	-	**< 0.001, 1.72**	**< 0.001, 2.00**	0.998, 0.13	**< 0.001, 2.77**	MD-5	Single-match week on Sunday
	MD-4	-	-	-	**0.007, 0.40**	**< 0.001, 1.78**	**< 0.001, 1.14**	MD-4	
	MD-2	-	-	0.275, 0.37	-	**< 0.001, 2.10**	**< 0.001, 1.65**	MD-2	
	MD-1	-	-	**< 0.001, 2.23**	**< 0.001, 2.85**	-	**< 0.001, 2.82**	MD-1	
	MD	-	-	**< 0.001, 1.44**	**< 0.001, 1.85**	**< 0.001, 3.09**	-	MD	

		MD-6	MD-5	MD-4	MD-2	MD-1	MD		
Condensed week	MD-6	-	0.899, 0.77	**< 0.001, 3.18**	0.163, 1.67	0.902, 0.65	**< 0.001, 4.42**	MD-6	Double-header week
	MD-5	-	-	**< 0.001, 3.54**	**0.010, 3.31**	0.293, 1.78	**< 0.001, 5.34**	MD-5	
	MD-4	**< 0.001, 3.19**	-	-	**< 0.001, 2.68**	**< 0.001, 3.00**	0.095, 0.60	MD-4	
	MD-2	**< 0.001, 1.50**	-	**< 0.001, 2.75**	-	0.749, 1.20	**< 0.001, 3.77**	MD-2	
	MD-1	**< 0.001, 3.06**	-	0.653, 0.46	**< 0.001, 2.58**	-	**< 0.001, 4.28**	MD-1	
	MD	0.846, 0.10	-	**< 0.001, 2.76**	**< 0.001, 1.37**	**< 0.001, 2.64**	-	MD	

*Abbreviations*: MD, match day; MD-1, day before match day; MD-2, 2 days before match day; MD-3, 3 days before match day; MD-4, 4 days before match day; MD-5, 5 days before match day; MD-6, 6 days before match day; HSR, high-speed running.

*Note*: Shaded cells show comparisons between days for Sunday and Double-header match schedules; bolded data indicate significant pairwise comparisons (*P* < 0.05).

### Saturday weekly match schedule

In weeks where one match was played on Saturday with at least seven days since the previous match, all load variables were significantly higher (*moderate*-to-*very large* effects) on MD than all days on which training sessions were conducted (MD-1, MD-2, and MD-4). Moreover, session duration and all external load variables were significantly lower (*small*-to-*very large* effects) on MD-1 than MD-2 and MD-4, while session-RPE was significantly lower (*moderate* effect) on MD-1 than MD-4.

### Sunday weekly match schedule

In weeks where one match was played on Sunday with at least seven days since the previous match, all load variables were significantly higher (*small*-to-*very large* effects) on MD than all days on which training sessions were conducted (MD-1, MD-2, MD-4, and MD-5). Moreover, session duration and all load variables were significantly lower (*moderate*-to-*very large* effects) on MD-1 than MD-2, MD-4, and MD-5, except for HSR distance and relative HSR distance which were significantly lower (*large*-to-*very large* effects) on MD-1 compared only to MD-2 and MD-4. In turn, significantly higher (*small*-to-*large* effects) relative total distance, HSR distance, and relative HSR distance were evident on MD-4 than MD-2 and MD-5, while significantly higher (*large*-to-*very large* effects) HSR distance and relative HSR distance were apparent on MD-2 than MD-5.

### Condensed weekly match schedule

In weeks where one match was played with only six days since the previous match, all load variables were significantly higher (*moderate*-to-*very large* effects) on days with matches (MD and MD-6) than all days on which training sessions were conducted (MD-1, MD-2, and MD-4). Duration was significantly lower (*moderate*-to-*very large* effects) on MD-1 than all other days (MD, MD-2, MD-4, and MD-6). Furthermore, all load variables, except session-RPE load, were significantly lower (*moderate*-to-*very large* effects) on MD-1 than MD-2.

### Double-header weekly match schedule

In the week where two matches were played, all load variables were significantly higher (*moderate*-to-*very large* effects) on days with matches (MD and MD-4) than all days on which training sessions were conducted (MD-1, MD-2, MD-5, and MD-6). In addition, significantly higher (*very large* effects) session-RPE and HSR distance were apparent on MD-2 than MD-5.

## DISCUSSION

This study is the first to comprehensively quantify the daily in-season loads encountered across weekly microcycles in the Australian national A-League Women’s competition. In providing novel, contemporary descriptive training and match load data for this population, some notable findings emerged. Key results included: (1) match days imposed the greatest daily loads with pronounced tapering on the day prior to matches across weekly schedules; (2) an extra training day was prescribed in single-match weeks when matches were played on Sunday compared to Saturday; and (3) weekly training microcycles were arranged similarly in condensed and double-header weeks compared to single-match weeks despite shorter turnaround times between matches.

While various match load variables have been reported for players competing in the A-League Women’s competition [13–17], no study has quantified the training loads experienced in this population. In this regard, the mean total distances covered during matches in each weekly schedule that we observed (7.4–7.9 km) were notably lower than those reported previously among players competing in this competition (8.7–10.0 km) [14, 15] and in the wider female soccer literature (9.6 ± 0.8 km) [33]. While these discrepancies in findings across studies may reflect variations between teams and/or seasons, they are likely due to previous studies only reporting data from players who participated in entire matches (i.e., 90 min of regulation playing time) and therefore had greater exposure to accumulate more distance as opposed to including data from players receiving less match exposure like bench and substituted players as we did (mean match durations of 69–79 min across schedules). In support of this notion, converting previously reported total distance data to relative values (i.e., per minute across 90 minutes) yields comparable relative total distance data to what we observed during matches (101–108 m · min^−1^vs. 97–111 m · min^−1^) [14, 15]. In contrast, the mean HSR distances covered that we observed during matches in each weekly schedule (0.5–0.6 km) were comparable to (0.6 km) [15] or lower than (2.5 km) [14] data reported in previous studies examining this population. Variations in findings across studies likely relate to the lack of standardization in methodologies to detect HSR, with previous work using speed thresholds of 12.2–19.0 km · h^−1^ [15] and 16–20 km · h^−1^ [14] compared to our threshold of > 18 km · h^−1^. In this regard, research [34] suggests that common HSR thresholds likely underestimate speeds that correspond with actual HSR in field-based team sports, supporting a higher threshold for this activity. In turn, the threshold in our study aligns with that collated across studies in a review examining female soccer players [28] and is closer to HSR thresholds adopted for female players by international soccer bodies (i.e., 19 km · h^−1^, Fédération internationale de football association [FIFA] and Union of European Football Associations [UEFA]) [35]. Indeed, the HSR distance we observed during matches falls within the range of values (0.1–1.7 km) reported in the female soccer literature using a standardized threshold of > 18 km · h^−1^ [28]. Nevertheless, multiple reviews have highlighted the lack of consistency in detecting HSR among female athletes competing in professional soccer [35] and wider field-based team sports [36], calling for more empirical evidence to inform development of standardized approaches moving forward.

When analyzing load periodization across days within our data, all load variables were greatest on match days and lowest on the day prior to matches, with the highest daily training loads generally administered two and/or four days before matches across all weekly schedules. These trends align with published recommendations for professional soccer teams [37], surveyed practices among strength and conditioning coaches (*n* = 45) working in elite French soccer academies [6], systematic evidence collated in male soccer players (*n* = 317) [38], and separate studies examining professional, female soccer competitions in Europe [8, 9, 11]. Consequently, it appears that weekly loading prescribed by the team we monitored competing in the A-League Women’s competition might be structured similarly to other high-level soccer competitions where a taper is applied leading into matches to optimize player readiness to perform and an acquisition period is applied mid-week eliciting suitable tactical, technical, physiological, and psychological stimuli to maintain or improve identified attributes of importance among players [37, 38]. However, some nuances in daily loading strategies emerged within our data when considering each of the match schedules.

Considering the single-match weeks in our study, the team we investigated prescribed an additional training session when the match was played on Sunday compared to Saturday, equating to ~54 min of additional exposure on average. Given players were generally afforded with seven days between matches in each of these schedules (i.e., same microcycle length), this pattern may reflect coaching preferences whereby MD-6 and MD-3 were consistently kept as rest days with similar training practices being delivered in the two days prior to matches regardless of when matches were played in single-match weeks. In turn, an additional rest day was administered during single-match weeks played on Saturday on MD-5. This approach appears to align with general practice evidenced by survey data [39] gathered from soccer practitioners (i.e., science staff, medical staff, and coaching staff) from various countries (*n* = 80) showing most use the first (70%) and/or second (51%) days following matches as rest days for player recovery. Of note, few surveyed practitioners (10%) used MD-3 for rest [39] as evident in our study, further emphasizing this arrangement may be specific to context or coaching preference. In this regard, using MD-3 for rest might have underpinned coaching decisions to elicit relatively consistent loads across MD-4 and MD-2 during these weekly schedules, which contrasts common load periodization schemes reported among soccer teams where MD-4 and MD-3 are typically the most demanding sessions outside of match days [6, 10, 11, 38].

Training periodization appeared to remain relatively consistent when microcycles between matches were reduced to six days in condensed weeks with rest days immediately following the initial match on MD-5 and on the customary MD-3. Surprisingly, more training (~43–55 min on average) was prescribed during the double-header week than single-match Saturday and condensed weeks, although this training was performed at lower external intensities (average relative total distance: 54.3 m · min^−1^ vs. 60.4–63.5 m · min^−1^; average relative HSR distance: 2.0 m · min^−1^ vs. 2.3–3.1 m · min^−1^) with a notable reduction in loading on MD-2 compared to MD-4. However, it should be noted that data were reported across all players who actively participated in each session and not differentiated according to playing role whereby reduced loading or additional rest may have been given to individual players who received greater match exposure during congested schedules [40]. Nevertheless, while these data are novel for female players competing in any soccer competition, relatively consistent daily loading schemes across weeks where one or two matches were played have also been reported in professional, male soccer players [21]. Indeed, survey data acquired from coaches working in elite English soccer settings (*n* = 94) suggest they consider the current match schedule only as *somewhat influential* when planning training (3.2 ± 1.2 on a 5-point Likert scale) [41]. Consequently, coaches may attempt to condense preparatory strategies into shorter timeframes within the week leading into matches when condensed or double-header weeks are faced without extensive consideration to the match schedule [21].

While this study is the first of its kind exploring female soccer players, providing foundation data for players competing in the Women’s A-League competition in a comprehensive, season-long analysis, notable limitations should be considered when interpreting the findings. Firstly, data are provided for the entire team while considering only match schedules. In turn, daily loads across weekly microcycles have been shown to differ according to wider player factors (e.g., position, role) [10, 42] and contextual factors (e.g., match location, match outcome) [43] in professional, male soccer players, which may have impacted our data but warrant further examination in professional, female soccer players. Secondly, we explored daily load fluctuations within different weekly match schedules; however, periodization strategies across longer timeframes, such as week-toweek load fluctuations, should be explored in future research given they have been shown to alter match loads among professional, male soccer players [44]. Thirdly, we provide an initial observational analysis of daily loads within the week among an A-League Women’s team. Future research exploring the effects of different daily loading strategies on match outcomes can extend on our findings to inform the development of optimal microcycle periodization strategies. Fourthly, our data are indicative of a single team across a season, generating few condensed (*n* = 3) and double-header (*n* = 1) weeks for analyses, which should be acknowledged when interpreting our findings. Consequently, our findings may not be indicative of loading prescribed across weekly microcycles in other teams within the A-League Women’s competition, with future research collaborations encompassing multiple teams and seasons in this competition encouraged for more robust evidence to be generated.

## CONCLUSIONS

This study provides new insights into the daily internal and external loads completed across the in-season under different weekly match schedules for players competing in the Australian national A-League Women’s soccer competition. In alignment with periodization strategies for weekly microcycles typically adopted in practice among various professional soccer competitions around the world [6–9, 11, 38], the greatest daily loads were encountered on match days with the lowest daily training loads apparent on the day prior to matches across the different weekly schedules. Novel to female soccer research on the whole, we observed that an extra training day was prescribed when matches were played on Sunday compared to Saturday in single-match weeks, while relatively similar periodization patterns were evident in condensed and double-header weeks compared to single-match weeks despite the increased congestion in scheduling. From a practical perspective, our findings provide a comprehensive understanding of daily loads encountered across a team competing in the A-League Women’s competition, which may be used as an initial reference for practitioners working in this competition or in wider women’s soccer settings. Furthermore, our data highlight that match scheduling may need greater consideration when prescribing training plans in this competition to ensure the augmented match loads, reduced recovery opportunity, and training needs of players are adequately balanced across congested weeks.

## Supplementary Material

Load distribution across weekly microcycles according to match schedule in a team competing in the Australian national A-League Women’s soccer competition
