# Supplementary material for: Load distribution across weekly microcycles according to match schedule in a team competing in the Australian national A-League Women’s soccer competition
Source: Biol Sport. 2024 Dec 13;42(2):265–77. doi: 10.5114/biolsport.2025.144413 (PMC11963116; doi:10.5114/biolsport.2025.144413)
Supplement: Load distribution across weekly microcycles according to match schedule in a team competing in the Australian national A-League Women’s soccer competition [file JBS-42-55029-s1.pdf]

SUPPLEMENTARY FILE. R SCRIPT USED FOR ANALYSES IN THIS STUDY.

```
library(readxl)
library(lme4)
library(dplyr)
library(lmerTest)
library(ggplot2)
library(multcomp)
library(officer)
library(effects)
library(sjPlot)
library(performance)
library(see)
library(patchwork)
library(flextable)

data_import < - read_excel("filename") #load data to r
data_import < - data_import % > %
filter(schedule %in% c("Saturday_normal", "Sunday_normal", "Sunday_double", "twogame")) #trial and internal matches were removed

#organise data and produce summary descriptives
summary_table < - data_import % > %
group_by(MD_code, schedule) % > %
summarise(
n_observations = n(),
mean_distance = mean(distance, na.rm = TRUE),
sd_distance = sd(distance, na.rm = TRUE),
mean_duration = mean(duration, na.rm = TRUE),
sd_duration = sd(duration, na.rm = TRUE),
mean_dist_per_min = mean(dist_per_min, na.rm = TRUE),
sd_dist_per_min = sd(dist_per_min, na.rm = TRUE),
mean_hsr = mean(hsr, na.rm = TRUE),
sd_hsr = sd(hsr, na.rm = TRUE),
mean_hsr_per_min = mean(hsr_per_min, na.rm = TRUE),
sd_hsr_per_min = sd(hsr_per_min, na.rm = TRUE),
mean_rpe = mean(rpe, na.rm = TRUE),
sd_rpe = sd(rpe, na.rm = TRUE),
mean_srpe_tl = mean(srpe_tl, na.rm = TRUE),
sd_srpe_tl = sd(srpe_tl, na.rm = TRUE))


#for model comparison only
full_model < - lmer(distance ~ MD_code + schedule + (1|player_id) + (1|Week), data = data_import)
interaction_model < - lmer(distance ~ MD_code*schedule + (1|player_id) + (1|Week), data = data_import)
separated_model < - lmer(distance ~ MD_code + (1|player_id) + (1|Week), data = sat_norm_data)
compare_performance(full_model, interaction_model, separated_model)
summary(full_model)
```

```
variables < - c("distance", "duration", "dist_per_min", "hsr", "hsr_per_min", "rpe", "srpe_tl") # List of variables
model_summaries < - list() # Create a list to store the model summaries
pairwise_results < - list() # Create a list to store the pairwise comparison results
# Loop through each variable and fit the model
for (variable in variables) {
formula < - as.formula(paste(variable, "~ MD_code + (1|player_id) + (1|Week)")) # Define the formula for the model using
                                                                                  the variable (separated_model)
model < - lmer(formula, data = data_import) # Fit the model using lmer function
model_summaries [ [variable]] < - tab_model(model, file = paste("Summary for", variable," separated_model.doc")) # Store the model
                                                                                                                    summary in the list

# Perform pairwise comparisons using Tukey's method
fit_tukey < - glht(model, linfct = mcp(MD_code = "Tukey"))
pairwise_results [ [variable]] < - summary(fit_tukey)
tab_model(model)

# Print the summary
print(paste("Summary for", variable, " separated_model "))
print(model_summaries [ [variable]])

# Extract residuals and create plots
residuals < - resid(model)
hist(residuals, main = paste(variable, "Residuals"), xlab = "Residuals")
qqnorm(residuals)
qqline(residuals)

# Print the pairwise comparisons
print(paste("Pairwise Comparisons for", variable, " separated_model "))
print(pairwise_results [ [variable]])

}
```
